# Mechanisms of cortical high-gamma activity (60-200 Hz) investigated with computational modeling

**DOI:** 10.1186/1471-2202-16-S1-P73

**Published:** 2015-12-18

**Authors:** Piotr Suffczynski, Nathan E Crone, Piotr J Franaszczuk

**Affiliations:** 1 Department of Biomedical Physics, University of Warsaw, Warsaw, 02-093, Poland; 2 Department of Neurology, The Johns Hopkins University School Of Medicine, Baltimore, MD 21287, USA; 3 Human Research & Engineering Directorate, U.S. Army Research Laboratory, APG, Aberdeen, MD 21005-5425, USA

## 

High-gamma activity (HGA) at frequencies 60-200 Hz have been observed during task-related cortical activation in humans [1] and in animals [2], and have been used to map normal brain function and to decode commands in brain-computer interfaces. To understand the role that HGA plays in both normal and pathological brain states, deeper insights into its generating mechanisms are essential. Because the neural populations recorded by LFPs and EEG cannot be comprehensively recorded at scales that are likely to be relevant, we used a biologically based computational model of a cortical network to investigate the mechanisms generating HGA. The computational model included excitatory pyramidal regular-spiking and inhibitory fast-spiking neurons described by Hodgkin - Huxley dynamics. We compared activity generated by this model with HGA that was observed in LFP recorded in monkey somatosensory cortex during vibrotactile stimulation. Increase of firing rate and broadband HGA responses in LFP signals generated by the model were in agreement with experimental results (see Figure 1).

**Figure 1:**
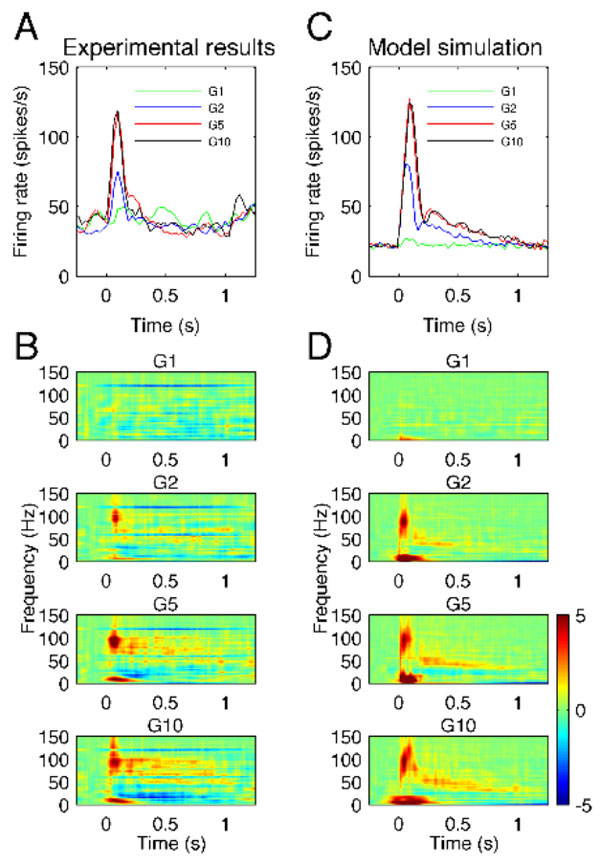
**Comparison of high-gamma observed *in vivo *(**AB**) and simulated in the model (**CD**) during sensory stimulation for different stimulus amplitudes denoted G1, G2, G5 and G10**. **AC**: average firing rate of neurons. **BD**: time - frequency maps of LFP signals.

## Conclusions

The HGA appear to be mediated mostly by an excited population of inhibitory fast-spiking interneurons firing at high-gamma frequencies and pacing excitatory regular-spiking pyramidal cells, which fire at lower rates but in phase with the population rhythm. HGA reflects local cortical activation under normal conditions and as such is a good candidate for mapping cortical areas engaged by a specific task. The mechanisms of HGA, in this model of local cortical circuits, appear to be similar to those proposed for hippocampal ripples generated by subset of interneurons that regulate discharge of principal cells.
